# The anti-plaque effect of high concentration sodium bicarbonate dentifrice on plaque formation and gingival inflammation, irrespective to individual polishing technique and plaque quality

**DOI:** 10.1186/s12903-023-03005-y

**Published:** 2023-05-11

**Authors:** Siren Abrahamsen, Odd Carsten Koldsland, Hans R. Preus

**Affiliations:** Department of Periodontology, Institute of Clinical Odontology, Faculty of Dentistry of Oslo, POB 1109, Blindern, Oslo, Norway

**Keywords:** Sodium bicarbonate, Dentifrice, Dental plaque, Inflammation

## Abstract

**Aim:**

To assess the anti-plaque effect of a high concentration sodium bicarbonate dentifrice on plaque formation, and gingivitis, as compared to a control toothpaste, irrespective of individual brushing technique and plaque quality.

**Methods:**

The experimental gingivitis model, with a split-mouth design was used to assess the anti-plaque effect of a high concentration sodium bicarbonate dentifrice on plaque formation. By producing individual fitted trays, the toothpaste was applied in the test quadrant and a control dentifrice in the contralateral. The participants used the individual fitted trays for 1 min every morning and evening, for 21 days. In this period, the participants was only allowed to brush the teeth in the opposite jaw, as usual. Twenty healthy individuals successfully completed the study.

**Results:**

At 21 days, there was no statistically significant difference between test quadrant and control quadrant with regard to plaque indices, gingival index and number of bleeding sites.

**Conclusion:**

This study demonstrated that the high concentration sodium bicarbonate dentifrice used did not produce statistically significant anti-plaque effect compared to the control dentifrice, in terms of Plaque- and Gingival Indices, number of bleeding sites or by Quigely and Hein, the Turesky modification Plaque Index, irrespective of brushing technique and individual plaque quality.

**Trial registration:**

Regional Committee for Medical Research and Ethics, South-East Norway in 2021 (REK.2021/370116).

Clinical Trial Registration: NCT05441371 (First registered 09/06/2022, First posted 01/07/2022) (http://www.clinicaltrials.gov). (Retrospectively registered).

## Introduction

A wide range of microorganisms colonize mucosal and dental surfaces to form well-structured biofilms [1]. Left undisturbed at, or below the gingival margin, an inflammatory response in the form of gingivitis or periodontitis will occur [2].

To prevent gingivitis, self-performed mechanical plaque removal is the key factor [3–5]. However, a systematic review and meta-analysis [6], reported that mechanical plaque-removal alone is not always sufficient to prevent the onset or recurrence of periodontal diseases suggesting that adjunctive chemical plaque control may be considered in specific cases.

Accordingly, a recent systematic review and network meta-analysis [7] assessed the efficacy of different oral hygiene products for chemical biofilm control. It was reported that even though all the tested products performed better than placebo, the effect was only statistically significant for those containing triclosan, stannous fluoride (SnF) and sodium metafluoride phosphate with zinc. Studies assessing sodium bicarbonate (NaHCO_3_) was not included. However, a systematic review and meta-analysis [8] concluded that the use of 67% NaHCO_3_ dentifrices could improve periodontal health in patients with gingivitis.

NaHCO_3_ was in 1903 recommended for “treatment of pyrrhea” [9] and has shown in vitro antimicrobial properties [10–12]. It was re-introduced in a dentifrice claiming that high concentration NaHCO3 toothpastes can improve periodontal health in patients with gingivitis, supported by a systematic review by Tascieri et al. [8]. The alleged mechanism of NaHCO3 stated by the producer (GlaxoSmithKlein, United Kingdom) is that it penetrates the layers of bacteria in the dental plaque, disrupts it and make it easier to remove.

Studies on NaHCO_3_ containing dentifrices typically test the dentifrices as part of the study-participant dental cleaning routine [8]. However, they do not consider that plaque quality differ between individuals [13], and that the individual brushing technique will affect the amount of plaque being removed [14], and are therefore not purely testing the suggested anti-plaque property.

Thus the aim of the present study was to evaluate the anti-plaque effect of a NaHCO_3_ containing dentifrice (Parodontax ultra clean™, GlaxoSmithKlein, United Kingdom) (Pdx) on plaque formation, and gingivitis, as compared to a control toothpaste, in a study design not affected by brushing technique and plaque quality of the individual participants, as well as the different abrasive qualities of the test and control dentifrice.

## Materials and methods

The experimental gingivitis model [2] was used to test the effect of Pdx on plaque formation, an experimental design widely used for registration of plaque and gingivitis in 21 days. The study was approved by the Regional Committee for Medical Research and Ethics, South-East Norway (REK.2021/370116). This study was registered at ClinicalTrials.gov (NCT05441371) and followed the ethical principles based in the Declaration of Helsinki and the reporting the criteria of the CONSORT guidelines.

The participants were bachelor- and master students at the Faculty of Dentistry, University of Oslo. These took special interest in the study and its carry-through since clinical research design is one of their subjects. Inclusion criteria comprised test persons 18 years or older, having at least three of the following teeth in contralateral quadrants; canine, premolars and 1^st^ molar with healthy gingiva, in quadrant one (test) and quadrant two (control). They also needed to sign an informed consent form. Exclusion criteria comprised: smoking, pregnancy, lactation, any chronic disease, clinical signs and symptoms of acute infection in the oral cavity, any prescribed or non-prescribed systemic or topical medication except oral contraceptives, use of antibiotics the last 3 months prior to the start of the study, history of alcohol or drug abuse and participation in other clinical studies in the last 4 weeks.

Twenty-five individuals volunteered for the study. Prior to baseline, three withdrew for personal reasons resulting in 22 participants signing the informed consent form. The participants were given a minor economic compensation (100 EUR) for the inconvenience. All information, administration and data collection were performed at the Department of Periodontology, Institute of Clinical Dentistry, University of Oslo, Norway.

At baseline, all participants received professional dental cleaning with rubber cup, pumice paste and dental floss, leaving the plaque score for every participant at zero. Individual fitted trays had been produced to fit the test- and control quadrants. Together with these, the participants received the test dentifrice (Pdx), and control, (Sensodyne Repair and Protect™, GlaxoSmithKlein, United Kingdom) and were instructed to apply the test dentifrice in one quadrant and the control dentifrice in the contralateral. Morning and evening, when the participants normally performed their oral hygiene routines, they first rinsed with water for 30 s. After placing the individual fitted tray, with its contents of test and control toothpaste, they upheld their regular oral hygiene regime in the opposing jaw where no registrations were done. After 1 min, the participants rinsed their mouth with water for 30 s. Then they removed the individual fitted tray and rinsed their mouth immediately with water for another 30 s. No dental cleaning, tooth pics or other, was allowed in these two, test and control, quadrants during the 21 days the experiment lasted. They repeated this procedure during the following 21 days. At day 7 and 14, the participants received a text message from the project manager with a short questionnaire about their adherence the protocol. All participants responded in kind, and no reminders had to be given. The use of individually fitted trays with their contents of test and control dentifrices secured that individual brushing technique, different biofilm qualities and abrasive qualities of the dentifrices were of no concern.

At day 21, one researcher (HRP) personally interviewed the participants about their adherence to the protocol, as well as complaints and subjective side effects. Plaque—(PlI) and Gingival Index (GI) [15] were registered, on 6 sites, i.e. first molar, premolars and canine in quadrant 1 (test) and quadrant 2 (control), by a periodontist (OCK). This was to avoid carry-over effects between the contents of the two individually fitted plastic trays in Q1 and Q2. Subsequently, a discoloration agent (Top Dent Rondell red, LIC Scandenta AS, Norway) was applied and photographs of the test and control quadrant were obtained. An individual, not associated with the study, mirrored a randomized 50% selection of the photographs, thereby blinding the examiners. With these photographs the Plaque Index of Quigley and Hein, the Turesky modification (TMQHPI) [16] was registered [17] by two blinded and previously calibrated examiners (OCK and SA). The two investigators did the evaluation separately both in time and place. One examiner (SA) did the evaluation twice with an interval of 21 days for intra examiner reliability purposes. The average value of the three scoring assessments for TMQHPI was used in the analysis. Following the scoring at day 21, the participants received professional tooth cleaning after ending the study.

### Statistics

The sample size calculation was based on similar studies, on the variable “average plaque score in each quadrant”, using a standard deviation of 0.4 [18], resulting in 20 test- and 20 control quadrants. Since we used a split-mouth design, 20 participants were needed to detect a mean difference in average plaque score of at least 0.4 between the test- and control quadrant with 80% power and a 5% significance level, at least 20 participants needed to be included. Some dropout was expected and for this reason, 25 individuals was initially invited in the study.

Shapiro-Wilks test and normal quantile plot was used to analyze normality of continuous variables. Primary outcome variable satisfied the normality assumption. Mean plaque score in the two quadrants was compared by using a two-sided independent sample t-test. As for the secondary outcomes, Wilcoxon signed-rank test was used to compare the mean difference between test and control, since the normality assumption was not satisfied. For both mentioned tests, significance level was set to 5%.

The inter- and intra-examiner agreement was evaluated by the use of Cohens Kappa statistics (k), using the tooth as the statistical unit.

For statistical analyses, the statistical Package for STATA (Stata version 17.0; college Station, TX, USA) was used.

## Results

**Fig. 1:**
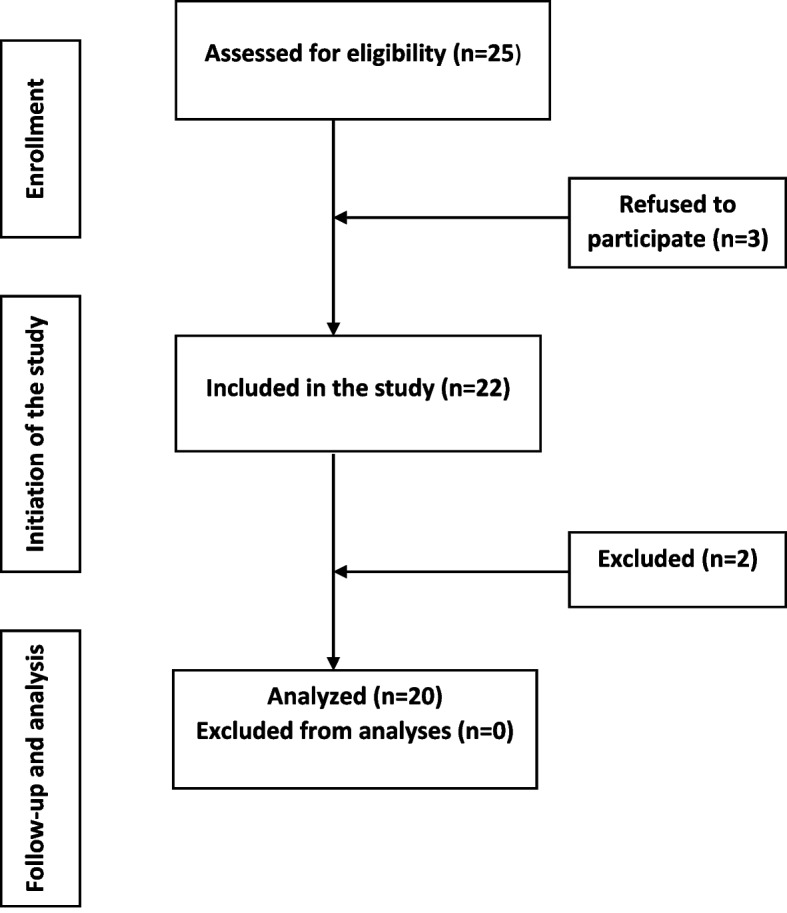
Flow chart

Twenty participants completed the full 21 days experimental period. Two participants was excluded from the study due to violation of protocol during the first week. Twenty participants had successful adherence to the protocol, still leaving the sample size large enough to satisfy sample size analysis and to obtain statistically significant results. A detailed flow chart diagram of patient recruitment is shown in Fig. 1.

The mean age was 26.6 (± 5.5) years and 68% were females.

A summary of mean values of main outcome and secondary outcomes are shown in Table 1.

**Table 1:**
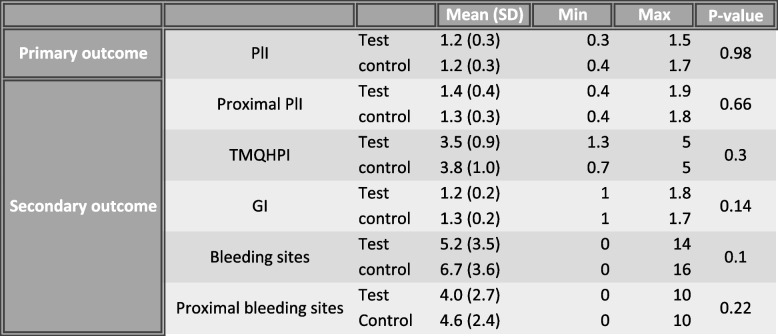
Descriptive statistics of test quadrant and control quadrant at 21 days follow-up

### Plaque Index, Gingival Index and bleeding sites

**Fig. 2:**
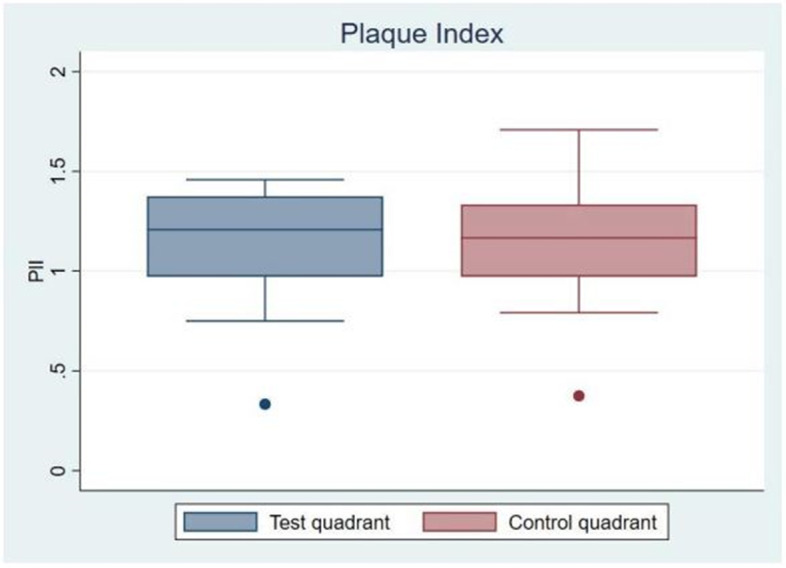
Box plot diagrams of the mean plaque score in test quadrant and control quadrant at 21 days follow-up

After 21 days, the test—and control dentifrice administered by individual fitted trays, resulted in a mean PlI of 1.2 ± 0.3 and 1.2 ± 0.3, respectively (*P* = 0.98), illustrated in Fig. 2, which shows the distribution of the mean plaque score in test quadrant and control quadrant. When only assessing interproximal surfaces the plaque index for test quadrant and control quadrant was 1.4 ± 0.4 and 1.3 ± 0.3, respectively (*P* = 0.66). Neither differences reached statistical significance.

The GI in test- and control quadrant was 1.2 ± 0.2 and 1.3 ± 0.2, respectively, leaving no statistically significant difference between the two quadrants (*P* = 0.14). The mean number of bleeding sites in test quadrant was 5.2 ± 3.5 as compared to control quadrant which produced a mean number of 6.7 ± 3.6 (*P* = 0.10). When only interproximal bleeding sites were assessed the mean number for test quadrant and control quadrant was 4.0 ± 2.7 and 4.6 ± 2.4 (*P* = 0.22), respectively. These inter-group differences was not statistically significant.

### Plaque Index of Quigley and Hein, the Turesky modification

Analyzing TMQHPI at 21 days, no statistically significant difference between test—and control dentifrice was found (3.5 ± 0.9 and 3.8 ± 1.0, respectively) (*P* = 0.3).

When assessing TMQHPI, the inter-examiner agreement varied from 75%-100% (k 0.6–1.0), regarded as substantial agreement to near perfect inter-examiner agreement. Intra-examiner agreement varied from 95%-100% (k 0.9–1.0), regarded as near perfect intra-examiner agreement.

No adverse side effects were reported. At day 21, nine out of 20 (45%) of the participants responded in the interview that they “did not like” the taste of the dentifrice.

## Discussion

This study assessed the effect of Pdx (NaHCO_3_) on plaque formation and gingivitis, as compared to a control dentifrice. The aim was to evaluate the anti-plaque effect of the dentifrice, in a study design that was not affected by brushing technique and plaque quality of the individual participants, as well as the different abrasive qualities of the test and control dentifrice. No statistically significant differences between the test and control on plaque formation or gingival inflammation was found.

It is well established that the individual brushing technique influence plaque removal [14]. None of the studies included in the review mentioned above [8] considered individual variations in brushing technique or plaque quality [19–25]. In the present study, the individual brushing technique could be disregarded since the test and control substance was introduced to the tooth surfaces by individually fitted trays for the participants and not by brushing. These were filled with test and control dentifrice leaving the only effect on plaque prevention and thus gingivitis from the chemical composition of the compound itself. In contrast, studies published by Yankell et al. [19] and Lomax et al. [23] had heterogonous test- and control groups at baseline, and the resulting change in plaque score in test- and control group at the end of the studies were small. The results may have been statistically significant but – as discussed in their paper—not clinically relevant.

The composition of dental plaque depends on a variety of competing microbial-microbial and host-microbial interactions [13]. For this reason, the composition and texture will vary between individuals. When conducting a parallel arm, clinical study, the individual plaque quality will affect the results when comparing test and control groups. The review published by Taschieri et al. [8] only include studies with this design [19–25]. The company providing the dentifrice was responsible for the randomization process in two of the studies [23, 24], and in one [20] the randomization process was not described. The main outcome in the present study was difference in plaque score between test- and control quadrants intra-individually at the end of the study. For this reason, the individual plaque quality could not bias results. To our knowledge, this is the first split-mouth clinical study assessing the efficacy the plaque inhibiting property of high concentration NaHCO_3_ dentifrice, irrespective of individual brushing technique, plaque quality and the abrasive effect of the dentifrice.

Ozaki et al. [21] compared a high concentration NaHCO_3_ dentifrice with one containing triclosan, with a 28 days follow-up period. Dentifrices containing triclosan have been reported to have significantly better effect on chemical biofilm control than placebo [7]. They used a triclosan containing dentifrice as a positive control and reported that both dentifrices showed significant reduction in plaque, and there was no statistically significant difference between test and control concerning GI. Mean GI at baseline in this study was 1.02, indicating minimal gingival bleeding. Short observation time and low to moderate grade inflammation at baseline makes it difficult to know if the effect was because of the pure effect of the dentifrice or the fact that the patients brushed their teeth better [26]. There are recommendations that the observation time should be 6 months or longer to reduce the risk of Hawthorne effect [27]. We will not challenge such a suggestion without scientific proof, but on the other hand, compliance from the test persons would be questionable, since 6 months without brushing teeth is not easy to overcome, as also would be ethical challenges concerning a protocol were the participants could not brush their teeth for 6 months. The observation time in the present study was similar as Ozaki et al. (21 days and 28 days respectively), but the use of the well-known and widely used experimental gingivitis model [2] in the present study makes up for this limitation.

The present study applied both PlI [15] and TMQHPI [16] to asses plaque formation. In the latter, a dental plaque disclosing agent was used which will stain both the biofilm and the pellicle [28]. Therefore, there was a risk of overestimation of the amount of biofilm on the dental surfaces. Also, the TMQHPI describes the spread of plaque on the dental surfaces, while gingivitis and bleeding mostly are affected by the plaque along the gingival margin which was registered by plaque index of Silness and Løe [15]. The studies [19–21, 24] included in the meta-analysis published by Tascieri et al. [8], only used TMQHPI, which in fact is normal for most plaque scoring studies. Although the PlI [15] would fully suffice in a study on plaque/biofilm and gingivitis, we also applied the TMQHPI [16] for comparison to these studies [19–21, 24], but emphasize that the spread of plaque/biofilm above the gingival margin is of less interest in studies on gingivitis.

Akwagyiram et al. [24] compared two dentifrices containing 67% NaHCO_3_ and 0% NaHCO_3_, respectively. When assessing TMQHPI, they evaluated interproximal score in addition to total score. An inter-group difference of 15% was observed in both total and interproximal assessment of TMQHPI. The reported difference of TMQHPI between the two dentifrices was significant, but small (0.46 and 0.45 for total and interproximal, respectively). They suggests that, “the plaque removing effect of NaHCO_3_ compared with brushing with water extends to areas in the oral cavity that are sheltered from mechanical effects of brushing teeth”. There might have been a significant difference, but the clinical relevance was inconclusive. In the present study, no such differences could be detected. When assessing both total and interproximal PlI, there was no statistically significant difference between test dentifrice and control dentifrice.

Recently, Tascieri et al. [8] published a systematic review and meta-analysis reporting; “the clinical use of 67% NaHCO_3_ toothpaste can improve periodontal health in patients with gingivitis”. Seven articles were included in the meta-analysis, with a follow-up ranging from 6 weeks to 6 months. There was high heterogeneity among the studies and four of the studies had moderate to high risk of bias. Moreover, the high content of sodium bicarbonate produce characteristic taste, which, when pursuing the effect in studies, will make blinding of patients difficult. To our knowledge, only two of the studies [19, 21] produced a control dentifrice with a similar characteristic taste as the test dentifrice. With the limited follow-up time of the included studies, one cannot rule out that the Hawthorne effect potentially could influence the outcome [26]. The seven studies included in the systematic review and meta-analysis had varying sample sizes. The companies providing the dentifrices being tested funded three of the studies included in the meta-analysis [22–24]. The other studies did not disclose if there were any funding or conflict of interest.

To our knowledge, Pdx sold in Europe does not contain SnF. However, during research it was discovered that SnF is an ingredient in Pdx sold in USA. Dentifrices containing SnF have demonstrated to have statistically significant better efficacy compared to placebo in regards of chemical biofilm control [7]. If studies done outside of Europe contain SnF, which can affect the results, it is important to disclose this information to the professionals and the public. This is especially important if this active ingredient is removed upon sales in different countries.

Pratten et al. [29] published an in vitro study showing that a 67% NaHCO_3_ slurry was able to change the morphology of a 14 day artificially produced and matured biofilm. In this in vitro study, saliva samples from 15 individuals was collected, and used to produce an inoculum, creating a biofilm on which they tested different concentration of NaHCO_3_ to assess the number of viable bacteria and the number of bacteria remaining on the substrata at different time intervals. They did not report on the other components of the slurry. As earlier mentioned, it has been reported that adding or removing different components may change the properties of a product [27]. However, In vitro results cannot be transferred to in vivo conditions, since the activity of the active ingredients may be different in vivo than in vitro [27]. To our knowledge, there are no reports about NaHCO_3_ having the same effect in vivo as in this artificially produced biofilm. It should also be mentioned the producer of a dentifrice containing 67% NaHCO_3_, sponsored the study [29].

The present study had some limitations. There was a limited sample size. The experimental gingivitis model was used and this justifies the short observation time, which is more a strength than a limitation since it allows the assessment of the true anti-plaque effect. Weekly assessment of compliance by directly contacting the participants could have been further assured by collecting the dentifrice tubes following day 21 for evaluation of compliance.

It is possible that placing and removing the individual fitted tray affect the amount of plaque, but since this will be the same for test and control; it will most likely not affect the results. We did not do microbial testing of the dental plaque, thus cannot say anything about potential changes in the bacterial plaque composition, but based on our results we did not find any statistically significant differences in any of the measured parameters.

## Conclusion

In conclusion, this study demonstrated that the use of Parodontax ultra clean™ dentifrice (NaHCO_3_) did not show a statistically significant anti-plaque effect compared to control dentifrice, in terms of PlI, GI, number of bleeding sites or TMQHPI, irrespective of brushing technique and individual plaque quality.

## Data Availability

The dataset is not publically available due to general data protection regulations but is available to corresponding authors on reasonable request.

## Abbreviations

NaHCO_3_: Sodium bicarbonate
TMQHPI: Plaque Index of Quigley and Hein, the Turesky modification
PlI: Plaque Index
GI: Gingival Index
Pdx: Parodontax ultra clean™, GlaxoSmithKlein, United Kingdom
SnF: Stannous fluorid
